# Full-Length SSU rRNA Gene Sequencing Allows Species-Level Detection of Bacteria, Archaea, and Yeasts Present in Milk

**DOI:** 10.3390/microorganisms9061251

**Published:** 2021-06-09

**Authors:** Isabel Abellan-Schneyder, Annemarie Siebert, Katharina Hofmann, Mareike Wenning, Klaus Neuhaus

**Affiliations:** 1 Core Facility Microbiome, ZIEL—Institute for Food & Health, Technische Universität München, 85354 Freising, Germany; isabel.abellan-schneyder@tum.de; 2 Chair of Microbial Ecology, ZIEL—Institute for Food & Health, Technische Universität München, 85354 Freising, Germany; annemarie.siebert@tum.de (A.S.); katharina.hofmann@tum.de (K.H.); mareike.wenning@lgl.bayern.de (M.W.); 3 Bayerisches Landesamt für Gesundheit und Lebensmittelsicherheit LGL, 85764 Oberschleißheim, Germany

**Keywords:** full-length sequencing, SSU rRNA gene sequencing, milk microbiota, LoopSeq

## Abstract

Full-length SSU rRNA gene sequencing allows species-level identification of the microorganisms present in milk samples. Here, we used bulk-tank raw milk samples of two German dairies and detected, using this method, a great diversity of bacteria, archaea, and yeasts within the samples. Moreover, the species-level classification was improved in comparison to short amplicon sequencing. Therefore, we anticipate that this approach might be useful for the detection of possible mastitis-causing species, as well as for the control of spoilage-associated microorganisms. In a proof of concept, we showed that we were able to identify several putative mastitis-causing or mastitis-associated species such as *Streptococcus* *uberis*, *Streptococcus* *agalactiae*, *Streptococcus* *dysgalactiae*, *Escherichia* *coli* and *Staphylococcus* *aureus*, as well as several *Candida* species. Overall, the presented full-length approach for the sequencing of SSU rRNA is easy to conduct, able to be standardized, and allows the screening of microorganisms in labs with Illumina sequencing machines.

## 1. Introduction

Sequencing has become a reliable and fast method over the years, allowing a time-efficient long-term screening perspective of bacterial communities from many different habitats, allowing the detection of potential pathogens. The 16S rRNA gene comprises nine variable regions (V-regions, V1–V9) that are separated by constant regions [1]. The more stable evolutionary constant regions are used for primer binding. The variable regions, evolved under varying evolutionary processes, are enclosed within a PCR product and are used for taxonomic classification and differentiation [2]. Today, the most frequently used method to study the microbiota of a given sample is short amplicon 16S rRNA gene sequencing, where one, two, or three adjacent variable regions of the 16S rRNA gene are sequenced on a short-read sequencer, e.g., Illumina’s MiSeq. The benefits of this technology are the low overall costs, the standardization of the protocols, and the ability to sequence in a high-throughput manner [3]. The drawbacks, on the other hand, are the read-length limitations of 600 bp maximum (due to the short-read sequencers) and the comparability issues of taxonomic profiles when using different short amplicon sequencing protocols or processing pipelines after the sequencing. 

Ideas on how to sequence full-length 16S rRNA genes using short-read sequencers like Illumina, together with assembly procedures, have been presented. For instance, Burke and Darling [4] and Karst et al. [5] described methods producing synthetic long-reads after the *de novo* assembly of fragmented 16S rRNA genes sequenced on a short-read sequencer. A similar approach was presented by Loop Genomic, a Silicon Valley-based company. The reconstruction of full-length molecules is possible after sequencing on a short-read sequencer. The key to their technology is to attach a unique molecular identifier (UMI) to each initial sequence at first, and to subsequently distribute this UMI intramolecularly. The latter is possible, most likely by the usage of a transposase (e.g., [6]) or circularization, followed by enzymatic digestion. 

Here, we used the Loop Genomic technology to improve the taxonomical classification down to the genus and species level, and compared this to short amplicon sequencing, either using the V1–V2 or V3–V4 regions. In short, the amplicon sequencing taxonomic resolution is more or less limited to the genus level. The sequencing of milk samples using short amplicon sequencing was performed intensively before, e.g., by Porcellato et al. [7], Taponen et al. [8], Metzger et al. [9], Cremonesi et al. [10], Metzger et al. [11], Pang et al. [12], Doyle et al. [13], Oultram et al. [14], Sokolov et al. [15] and Li et al. [16]. Nevertheless, full-length sequencing approaches are rare and, if published, are mostly performed using long-read sequencers, e.g., Catozzi et al. [17]. In a proof of concept, we assessed whether we could identify putative mastitis pathogens at the species level in random bulk-tank milk samples. We believe better species resolution to be of interest for several other cases, e.g., detecting potential pathogens in milk, or detecting contamination with specific spoilage-related bacteria in dairy products.

As said, we used bovine bulk tank milk samples, but also two mock communities of known composition. The mock communities were included in order to precisely assess the known taxonomic profile of a given sample, and to allow us to draw conclusions about each method. Using the milk samples, we showed the feasibility of the detection of potential pathogens at the species level. In the past, different genera or bacterial species were found to be associated with mastitis, which is defined as an inflammation of the mammary gland [18]. The species most frequently associated with mastitis are non-aureus *Staphylococci*, *Streptococcus uberis*, *Streptococcus agalactiae*, *Streptococcus dysgalactiae*, and also *Staphylococcus aureus*, *Corynebacterium bovis*, *Escherichia coli* and *Klebsiella pneumoniae* [19,20,21,22,23]. Thus, the objectives of this study were (i) to compare short amplicon with full-length 16S rRNA gene sequencing concerning species identification and (ii), as proof-of-concept, to further assess whether we could detect some of these bacteria at the species level in our data set from the milk samples. Furthermore, due to the use of a primer mixture that targets all small subunit (SSU) rRNAs, bacteria, archaea and yeasts were detected at the same time. This enables the collection of additional information of archaeal and eukaryotic microorganisms in the milk microbiota, as these groups of microorganisms are typically not targeted by short amplicon sequencing, which is directed mainly towards bacterial 16S rRNA genes.

## 2. Materials and Methods

### 2.1. Milk Samples

Between May and June 2020, the bulk-tank raw milk of different German farms was collected by two dairies and conserved with azidiol (0.33 % *v*/*v*) automatically by the collection trucks. Ten milk samples, each representing a different farm, were selected randomly from farms in Southern Germany participating in a raw milk research project supported by the German Federal Ministry for Food and Agriculture (project number: 281A105616). The dairies providing the milk samples also provided information on the total bacterial counts (measured as colony-forming units, CFU/mL) and the average values of the somatic cell count (SCC) recorded within the last 12 months for each farm. The individual bacterial counts (IBC/mL) were detected via flow cytometry using a BactoCount IBC (Bentley Instruments EU, Maroeuil, France). Sample vials, containing 30–40 mL conserved milk, were shipped and stored refrigerated for a maximum of three days until processing. Of the above-mentioned project, we used the milk sample numbers 796, 797, 798, 879, 880, 978, 979, 980, 982 and 983. Further characteristics of the used raw milk samples were recorded beforehand (Table 1).

### 2.2. DNA Extraction of the Bovine Milk 

Cells were harvested and DNA was extracted as previously described by Siebert et al. [24]. In brief, 30 mL bovine raw milk were treated with 1.8 mL 0.3 M EDTA. After the cell harvesting by centrifugation (20 min at 4 °C) and the removal of the milk fat and skimmed milk in the supernatant, the selective lysis of the somatic DNA was performed using proteinase K (20 mg/mL, AppliChem GmbH, Darmstadt, Germany) and DNase I (Thermo Fisher Scientific, Waltham, MA, USA). The DNA extraction was performed with the PowerFood Microbial DNA isolation kit (Qiagen, Hilden, Germany), modified by an additional enzymatic lysis step. Towards this end, lysozyme (25 µg/mL, Carl Roth) and mutanolysin (100 U, Sigma-Aldrich, St. Louis, MO, USA) were added to the cell suspensions together with the MBL solution of the DNA isolation kit, followed by an incubation at 37 °C and 350 rpm for 30 min. After an additional treatment with proteinase K (12.5 mg/mL, AppliChem), the remaining bacterial cells were disrupted in tubes with silica beads using a FastPrep-24TM instrument (MP Biomedicals, LLC, Irvine, CA, USA). The subsequent DNA isolation followed the manufacturer’s protocol, i.e., that of the PowerFood Microbial DNA isolation kit. The DNA was finally eluted in 2 × 24 µL of PCR-grade water (preheated to 55 °C). 

### 2.3. DNA Extraction of the Mock Communities 

The Zymo mock community was purchased as ZymoBIOMICS Microbial Community Standard (D6300, Zymo Research Europe GmbH, Freiburg, Germany) and the DNA was extracted by an adapted protocol originally described by Godon et al. [25], slightly modified. The details can be found in Reitmeier et al. [3]. The ZIEL2 mock community was prepared and extracted as described in Abellan-Schneyder et al. [26]. Briefly, 19 bacterial strains (18 different bacterial genera) of diverse taxonomy were cultured and harvested afterward by centrifugation. A genomic DNA (gDNA) extraction was performed separately for each strain. The ZIEL2 mock community was constructed by pooling 12 ng of each bacterial gDNA.

### 2.4. Short Amplicon 16S rRNA Gene Library Preparation 

For the amplification of the V1–V2 and V3–V4 regions of the 16S rRNA genes, 2-step PCRs of 20 and 10 cycles for the milk samples and 15 and 10 cycles for the mock communities were performed as described in Table S1 (first step PCR), Table S2 (second step PCR) and Abellan-Schneyder et al. [26]. 

For V1–V2, primers 27F and 338R [27] and for V3-V4, primers 341F and 785R [28] were used (Table S3). Further details and work time estimations can be found in Reitmeier et al. [3]. 

### 2.5. Library Quality Check and the Sequencing of the Short Amplicons 

The concentrations of the final PCR products were measured in triplicates using a Qubit 4.0 (Thermo Fisher Scientific, Waltham, MA, USA). Each sample was adjusted to 0.5 nM. All of the samples were pooled and sequenced in the paired-end mode for 2 × 300 bp (PE300) using a MiSeq system (Illumina, Inc., San Diego, CA, USA), following the manufacturer’s instructions. The final DNA concentration of the library was 12 pM, and 15% (*v*/*v*) PhiX was added. 

### 2.6. Synthetic Long-Read Sequencing Using the LoopSeq 16S & 18S Microbiome Kit

The library preparation was performed as described by LoopGenomics for the LoopSeqTM 16S & 18S Long Read Kit (Version 2.1, Loop Genomics, San Jose, CA, USA). This kit enables us to generate de novo assembled long reads which are able to be sequenced on a short-read sequencer. The de novo assembly is possible due to the intramolecular distribution of a specific unique molecular identifier (UMI), which is unique for every initially tagged full-length molecule. At the same time, the full-length PCR products are split into shorter fragments that can be sequenced using short reads. The exact mechanism of the LoopSeq protocol is proprietary, but possibly relies on a transposase-like enzymatic function. Furthermore, the LoopSeq kit contains primers which can bind to 16S and 18S rRNAs, allowing the estimation of bacteria and archaea together with eukaryotic microorganisms. In brief, 5 µL 1:10-diluted gDNA (in 10 mM TRIS buffer, pH 8.5) was used for the ‘Enrichment’. The enrichment PCR was performed for 30 cycles of denaturation, annealing and elongation, as described in Table S4. Instead of using the pre-mixed enrichment primer from the supplier, a custom 2 µM primer mix was used. We ordered four forward primers and two reverse primers, HPLC purified, in 10 µM stock concentrations (biomers.net GmbH, Ulm, Germany). Every primer was diluted with water to 2 µM and added to the final primer mix in equimolar ratios. The sequences of the six different primers were taken from the Loop Genomics primer file ‘Genome Amplification Oligonucleotide Sequences’, Version 1.0, which is available from Loop Genomics. Concentrations of the 1:10 diluted PCR products (i.e., ‘Enrichment’) were measured using a Qubit. For all of the samples, concentrations >0.1 ng/µL could be detected after the PCR. Barcodes were assigned to each sample as described by the manufacturer. The barcode calibration was performed in a total volume of 10 µL instead of 20 µL. Based on the calculated sample concentration, the samples were diluted to about 8,000 barcodes per sample. The barcode distribution was performed according to the manual, but concentrations of each sample were assessed using a Qubit before the pooling. The samples were pooled in equimolar amounts based on the PCR-product concentrations. Afterward, the pool was purified using AMPure XP Beads according to the manual. Next, the barcode distribution and library preparation were performed as described in the LoopSeq manual. In this work, Index Primer 1 was used for the pool. The final pool was loaded on an Agilent HS DNA Chip to assess its quality. An average library size of 600 bp was determined and deemed adequate for sequencing. The pool was adjusted to a concentration of 2 nM and prepared for sequencing on an Illumina MiSeq as described by the sequencer’s manual. The final library concentration was 12.5 pM, and 5% PhiX was added. The raw short reads were uploaded to the LoopGenomics platform. The results, including the assembled full-length sequences, stats and taxonomy files, could be downloaded from the website after a run time of about 2 h. 

### 2.7. Data Analysis of the Short Reads Using DADA2

The primer sequences of the short amplicon reads were trimmed using cutadapt [29]. Afterward, the samples were processed with the DADA2 pipeline v1.18.0 [30]. The following options were used: paired-end mode, a truncation length of 200/180 bp for V1–V2 and 260/220 bp for V3–V4, maxN = 0, maxEE = 2/2, and trunQ = 2. As the reference database, SILVA v132 (https://www.arb-silva.de/documentation/release-132/, accessed on 2 December 2020) was chosen.

## 3. Results

### 3.1. Protocol Overview for the Short and Full-Length 16S rRNA Gene Sequencing

**Figure 1:**
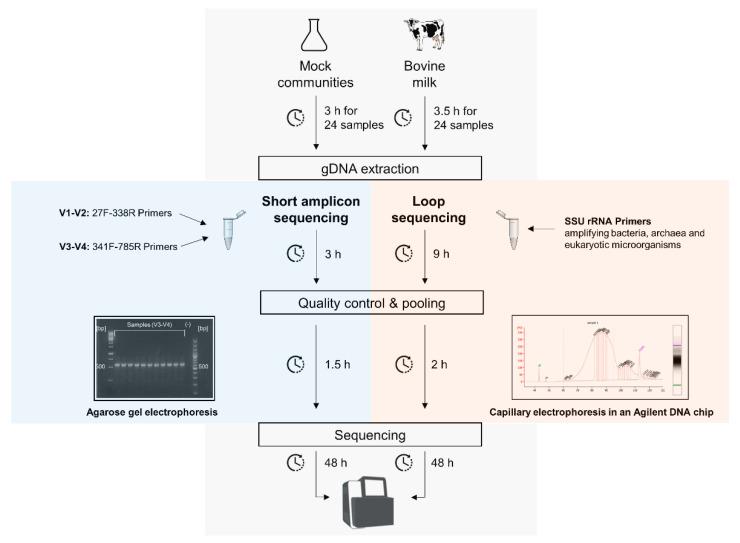
Overview of the experimental procedure. Mock communities of known composition and bovine raw milk samples were used for the microbial gDNA extraction (grey above). The samples were prepared for short amplicon 16S rRNA gene sequencing (blue) and full-length sequencing using the 16S/18S kit targeting the SSU rRNA (orange). After the libraries were prepared, cleaned, and attested to be of good quality, the sequencing was performed on an Illumina MiSeq (grey, below). The execution time estimations in hours are shown for all steps.

In the present study, we sequenced ten bovine raw milk samples and two mock communities of known composition (Figure 1). The isolated DNA was sequenced on the one hand using short amplicon primers spanning the V-regions V1–V2 and V3–V4 of the 16S rRNA genes (highlighted blue in Figure 1). On the other hand, the samples were processed by using the LoopSeq™ 16S & 18S Long Read Kit (Loop Genomics, San Jose, California, USA, highlighted orange in Figure 1). 

### 3.2. Performance on Mock Communities 

**Figure 2:**
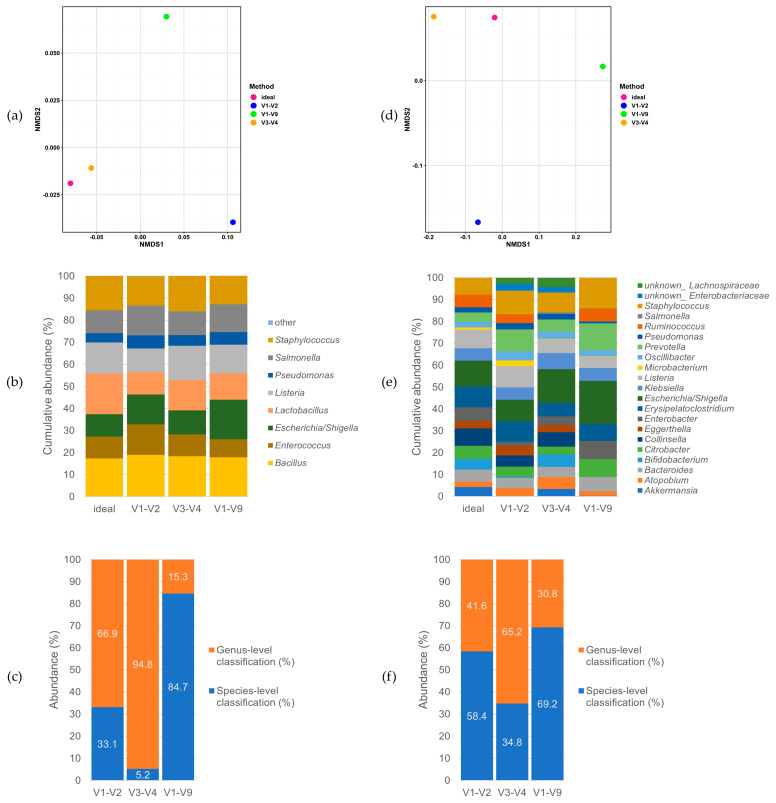
Global performance of three different sequencing procedures using two different mock communities: Zymo (**a**–**c**) and ZIEL2 (**d**–**f**). (**a**) NMDS plot showing the results for the Zymo mock community sequenced with V1–V2 (blue), V3–V4 (orange), and V1–V9 (green). Furthermore, the ‘ideal’, i.e., the theoretical composition of the Zymo mock is added as an additional data point (violet). (**b**) The relative abundance of bacteria included in the Zymo mock, consisting of eight bacterial genera (and two eukaryotic, not shown). The first column shows the ideal (theoretical) composition, while the following columns show the data gained for V1–V2, V3–V4 and V1–V9. (**c**) Overview for the percentage of the bacteria of the Zymo mock, which could be classified down to the species- (blue) or only to the genus-level (orange) for the three sequencing procedures (V1–V2, V3–V4, and V1–V9). As can be seen, the full-length approach (V1–V9) outperformed the short amplicon approaches (V1–V2 and V3–V4) in species classification. (**d**) NMDS plot showing the results for the ZIEL2 mock community sequenced with V1–V2 (blue), V3–V4 (orange) and V1–V9 (green). Further, the ‘ideal’, i.e., the theoretical composition of the Zymo mock is added as an additional data point (violet). Please note the larger differences between the data points indicated by the scale compared to panel **a**. (**e**) Relative abundance of bacteria included in the ZIEL2 mock, consisting of 18 bacterial genera. The first column shows the ideal (theoretical) composition, while the following columns show the data gained for V1–V2, V3–V4, and V1–V9. The different sequencing approaches lead to wider differences when comparing the relative abundance in each case to the ‘ideal’ composition. This was already reflected in the larger distances in the NMDS plot in panel **d**. (**f**) Overview for the percentage of the bacteria of the ZIEL2 mock community, which could be classified down to the species- (blue) or only to the genus-level (orange) for the three sequencing procedures (V1–V2, V3–V4 and V1–V9). The full-length approach classified more reads correctly down to the species-level than the short amplicon sequencing (V1–V2 and V3–V4) approaches.

Two short amplicon procedures (V1–V2 and V3–V4) and the full-length 16S & 18S long read method (basically including V1–V9 concerning 16S rRNA genes and, thus, designated as such from hereon) were compared using mock communities at first (Figure 2). When analyzing only the less-complex Zymo mock community, which consists of eight different bacterial genera, the distances in the non-metric multidimensional scaling (NMDS) plots were relatively low (Figure 2a, compare the scale to panel d). Generally, distances are considered to be excellent (e.g., in the sense of being negligible) when they are <0.05. Otherwise, they are considered good at <0.1, usable at <0.2, and not acceptable at ≥0.2, i.e., in the sense that the results are ‘too different’ [31]. These low distances observed here for the Zymo mock were also reflected in the resulting taxonomical profiles (Figure 2b), showing no major deviations between any method used and the ideal theoretic composition. Overall, the Zymo mock performed satisfactorily for all of the sequencing approaches. However, naturally occurring communities are normally poly-species. Thus, the more complex ZIEL2 mock community, which consists of 18 different genera, was used. Here, the results between the different methods deviated more. Even though V3–V4 and the ideal composition showed short distances, the distances to the ideal composition were larger for V1–V2 and V1–V9 (Figure 2d). This finding was reinforced when the taxonomic profiles were analyzed and compared (Figure 2e). For V1–V2, *Akkermansia*, *Bifidobacterium* and *Enterobacteria* were underrepresented compared to the ideal composition. For V3–V4, *Ruminococcus* and *Microbacterium* were extremely reduced in proportion compared to the ideal mix. Concerning V1–V9, no identification of *Bifidobacterium* and *Collinesella* was possible at all, and a dramatic underrepresentation of *Akkermansia*, *Eggerthella* and *Microbacterium* was observed. Nevertheless, one of the key features of full-length approaches is a species-level classification. Therefore, we analyzed the precision of the species-level classification for each of the different sequencing approaches. For the Zymo mock community, we found that nearly 85% of all reads could be identified down to the species level when using V1–V9. In contrast, only 33% and 5% for V1–V2 and V3–V4, respectively, could be classified at the species level (Figure 2c). 

The picture changes when we use the more complex ZIEL2 mock. For this mock community, V1–V9 also performed best, allowing 69.2% of the communities to be identified down to the species level. Interestingly, V1–V2 also did relatively well, allowing up to 58.4% of the bacteria to be identified to the species level. As before, V3–V4 performed worst, i.e., only 34.8% of the species could be identified here (Figure 2f).

### 3.3. Performance on the Bovine Milk Samples for Bacteria Identification 

Comparisons of the LoopSeq results with short amplicon profiles were only possible for bacteria, as the short amplicon primers which were used were not optimized for the targeting of archaea and were not suitable for eukaryotes. The five genera/species with the cumulative highest amount of reads for bacteria in the raw milk samples sequenced using V1–V9 (LoopSeq) were *Romboutsia* sp., *Turicibacter* sp., *Paeniclostridium* sp., *Clostridium* sp. and *Streptococcus uberis*. For comparison, in V1–V2, the five bacterial genera with the most reads were *Corynebacterium*, *Romboutsia*, *Streptococcus*, *Turicibacter,* and *Staphylococcus*, and for V3–V4, these were *Romboutsia*, *Streptococcus*, *Corynebacterium*, *Turicibacter* and *Paeniclostridium*. 

**Figure 3:**
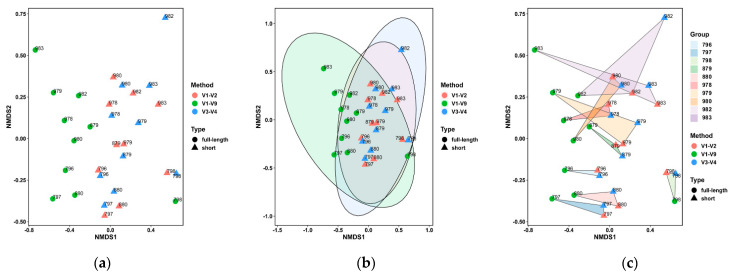
Non-metric multidimensional scaling (NMDS) plots for the comparison of the sequencing results of V1–V2, V3–V4 and V1–V9. The NMDS plots highlight that full-length sequenced samples cluster a little apart from V1–V2 and V3–V4 sequenced samples (**a**). This becomes more evident when samples are grouped by targeted region (**b**). However, differences between sequencings of the same sample using different primer pairs are large and are not only explainable through the targeted region (**c**).

When analyzing the 50 most prevalent bacterial genera, clustering was not significantly dependent on the targeted V-region or sample origin (Figure 3a). However, the V1–V9 sequenced samples seemed to cluster apart from V1–V2 and V3–V4, which partially overlapped in the clustering and showed overall smaller clustering distances (Figure 3b). Nevertheless, when focusing on each sample analyzed with different methods (e.g., V1–V2, V3–V4 and V1–V9) in the NMDS plot, it becomes clear that changing the method leads to sometimes extensive intra-sample distances (Figure 3c). 

**Figure 4:**
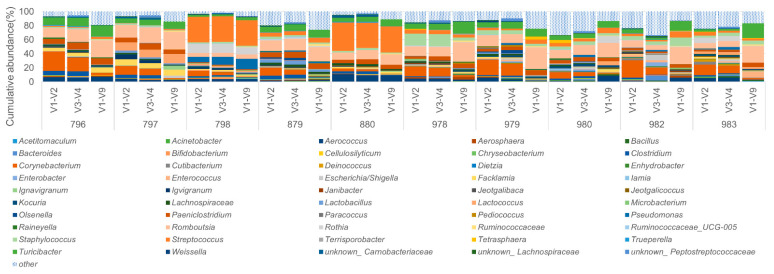
LoopSeq and short amplicon sequencing compared at the genus level for the top 50 bacterial genera. The relative abundances (%) at the genus level of the 10 raw milk samples sequenced by each method (V1–V2, V3–V4 and V1–V9) vary from each other (between samples from different origins) when the 50 most abundant taxa are analyzed. All other remaining taxa are shown in hatched blue.

Regardless of the above, taxonomic profiles showed recurrent similarities when the same sample was analyzed (Figure 4). For example, milk samples 798 and 880 had a higher amount of *Streptococcus* (rich orange) compared to the other samples.

### 3.4. Identification of Archaea and Eukaryotes in Bovine Milk Samples Using the LoopSeq Protocol

**Figure 5:**
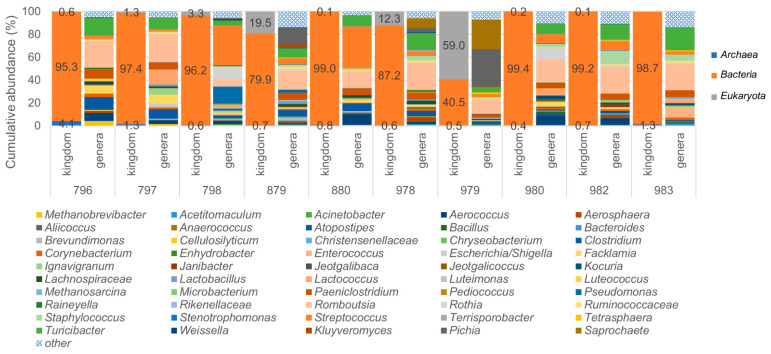
Relative abundance (%) at the kingdom and genus levels after the 16S/18S rRNA (SSU) gene sequencing analysis using the LoopGenomics kit. Taxonomic classification regarding the kingdom (**left**) and genus-level of the top 50 taxa (**right**) of each LoopSeq-sequenced milk sample. All of the other remaining taxa are shown in hatched blue.

Most interestingly, when analyzing the samples using the 16S & 18S Long Read Kit, referred to as V1–V9 before, not only bacteria but also archaea and eukaryotes could be identified. When analyzing the taxonomical composition at the kingdom level, sample 979 was shown to have the highest number of reads mapping to eukaryotes, and sample 796 had the highest number of archaeal reads (Figure 5). Nevertheless, the amount of eukaryotic and archaeal contribution to the raw milk microbiota was mostly low except for samples 796, 879 and 979. Thus, the milk samples were shown to be diverse and have unique taxonomic profiles. 

In total, we were able to identify 41 different eukaryotic genera and 52 eukaryotic species, as well as seven archaeal genera and seven different archaeal species in the milk samples tested. For eukaryotes, *Pichia* (54.2%), *Saprochaete* (39.5%) and *Kluyveromyces* (2.9%) were by far the most dominant genera, whereas for archaea, these were *Methanobrevibacter* (80.6%) and *Methanosarcina* (16.7%). The overall top five hits in either kingdom are listed in Table 2. 

### 3.5. Identification of Putative Mastitis-Causing Pathogens

Concerning archaea, *Methanobrevibacter*, *Methanocorpusculum, Methanosphaera* and *Methanomassiliicoccus* are the most common archaeal bovine-associated genera [32,33]. Of those, we could identify *Methanobrevibacter*, *Methanocorpusculum,* and *Methanosphaera*. For archaea, no association with mastitis is known. In contrast, for eukaryotes, *Cryptococcus neoformans*, *Candida albicans* and other *Candida* species are known to be potential mastitis-causers [34]. A cultivation-based study from 2008, which analyzed Brazilian subclinical mastitis milk samples, found that *Candida*, *Pichia*, *Cryptococcus* and *Rhodotorula* were the most frequent genera. However, they could not be directly linked to the subclinical mastitis state in the mentioned publication [35]. Regarding *Cryptococcus* and *Candida*, we were unable to identify *Cryptococcus* reads in any one of the milk samples, but for *Candida,* we identified *Candida boidinii*, *Candida metapsilosis*, *Candida intermedia*, *Candida zeylanoides* and *Candida pararugosa*. 

Next, it was investigated whether putative mastitis-causing bacteria could be identified down to the species level. Towards this end, it was checked whether the species listed as mastitis-causing pathogens by Cobirka et al. [20] could be found in our dataset (Table 3). From the 25 genera/species listed by Cobirka et al. [20], we could identify 19 here. Admittedly, five species (*Enterococcus durans*, *Enterococcus faecium, Klebsiella oxytoca, Serratia* spp. and *Staphylococcus simulans*) were only identified in very low amounts of <10 reads.

## 4. Discussion

### 4.1. Full-Length SSU rRNA Gene Sequencing Improves Species-Level Classification but Shows Primer Issues

A variety of different factors can bias sequencing approaches targeting the 16S rRNA gene. The most well-known and studied are the sample collection, stabilization and transport to the laboratory, DNA extraction method, DNA concentration, primers targeting different V-regions, PCR condition and settings, laboratory practice, analysis pipelines, and reference databases [24,26,36,37,38,39]. In our results, it became obvious that, e.g., primer pairs 27F and 338R underrepresent *Akkermansia*, whereas this is not the case for 338F and 785R. Because the LoopSeq kit uses the forward 27F primer, low amounts of *Akkermansia* and *Bifidobacterium* were expected, similar to V1–V2, which also used the 27F-region for priming. However, the failure of the detection of *Collinsella* and *Eggerthella* in the LoopSeq results could not be explained by using this particular forward primer. In contrast, it was shown in a previous study that the use of primer pair 1115F and 1492R led to an underrepresentation of those two genera [26], which might suggest that the reverse primer 1492R is responsible for this inferior result concerning the latter two genera. This highlights that studies must carefully test whether the primers used are suitable for the expected results. Furthermore, improved full-length primer pairs or strategies such as the rRNA full-length approach of Karst et al. [5] are needed. The method of Karst et al. is primer-independent because it starts from the actual rRNA molecule, which is reverse transcribed in cDNA, and not from rRNA genes. 

Overall, we suggest that the LoopSeq approach could be improved by enhancing the 16S and 18S targeting primers. For instance, the current forward primer 1 is the commonly used 27F-CM. It has already been shown [40] that this primer poses three mismatches when *Bifidobacteria* should be amplified, and thus shows a decreased binding towards the 16S rRNA genes of this genus. Accordingly, we saw a dramatic underrepresentation of *Bifidobacteria* in the ZIEL2 mock (Figure 2E). Perhaps the primer mix should include further or other 27F-based primers (e.g., 27F-YM) that are improved in bacterial targeting.

We want to emphasize a well-thought-out study design and the need for sufficiently complex mock communities, as we have shown that the Zymo mock is too simple to illustrate possible biasing effects (Figure 2). This becomes even more important when low biomass samples are analyzed. It was previously shown that milk samples are of low bacterial biomass, and are therefore prone to be contaminated by bacteria from the environment [27,38,41]. Thus, controls and mock communities of sufficient complexity should always be sequenced in order to secure the reliability of a study. Eisenhofer et al. [42] published a well-thought-out list of several methods which can be used to minimize the influence of contaminant DNA on low bacterial biomass samples that should be taken into account. To name a few, the use of controls, suitable protective clothing including gloves, masks and clean suits, decontamination and cleaning steps, and protection steps during the sample processing like the use of unique barcodes should be considered [42]. 

We showed that by using a full-length SSU rRNA sequencing method, species-level identification clearly is increased (Figure 2). Nonetheless, to further improve species-level classification, we see a need for higher resolution environment-specific databases, such as those described by Dueholm et al. [43] and Escapa et al. [44], allowing precise taxonomic comparison and classification. Escapa et al. could, for example, show that the training of the Ribosomal Database Project (RDP) classifier using a habitat-specific training set improved the taxonomical assignment for short- as well as long-read sequences at the species-level. Such bioinformatical developments, besides methodological improvements (e.g., using full-length strategies), will increase the overall amount of reads which can be classified down to the species level. Improvements in species-level classification have also been shown by Jeong et al. [45] for the use of the LoopSeq approach on human fecal samples. Furthermore, for those samples, the taxonomic resolution was improved when compared to short amplicon V3–V4 protocols while analyzing *alpha*-diversity, relative abundance frequency and identification accuracy.

### 4.2. Using Full-Length Sequencing Approaches for Microbial Monitoring

Microbial monitoring using sequencing-based approaches can facilitate and allow for the detection of possible contaminants in food samples. Thus, we assessed, in a proof-of-principle, whether we could detect putative mastitis pathogens in our sample set. Importantly, in our bulk tank milk samples, we found 17 out of 25 listed putative mastitis-causing bacteria in the full-length data [20]. For instance, *S. uberis* was found in the highest amounts among the analyzed species. However, most of the reads were contributed by three samples (880, 504 reads; 978, 422 reads; and 983, 422 reads), which made up 80% of all of the combined *S. uberis* reads found in all ten milk samples. Thus, we believe that a future study including samples of known (sub-)clinical mastitis cases is of further interest. Concerning *S. uberis,* for example, it is yet unclear which amount might be tolerated. Possibly, a threshold in either relative or absolute abundance, or the relative abundances between different mastitis-causing bacteria (i.e., a dysbiotic state) must be defined for these organisms. Mastitis-causing pathogens are often found to be opportunistic and, therefore, the mere presence of those species currently does not allow us to draw conclusions about a possible state of inflammation or disease. Nevertheless, full-length SSU rRNA gene sequencing allows relative quantifications, which could be used to determine dysbiotic states. 

As our study design was intended to be a proof-of-principle concerning a species-level detection of potentially pathogenic bacteria in milk, we assessed whether we could find such species in our dataset. However, these bacteria could also be simple contaminants, as previously reported [12,14,46,47,48,49]. Nevertheless, in many of those previously performed studies, short-amplicon sequencing strategies were applied targeting either V1-V2, V3-V4 or V4 alone. These studies are, therefore, limited in their taxonomic resolution. In contrast, targeting the full-length SSU rRNA gene helps to identify bacteria, archaea, and eukaryotes at improved taxonomic levels. For instance, Catozzi et al. [17] published a full-length 16S rRNA sequencing strategy for the milk microbiota of water buffalos. In this study, the authors demonstrated that full-length strategies are suitable for species-level detection. Nevertheless, their strategy had some drawbacks, such as, for instance, a higher error rate of the reads obtained by using a MinION sequencer, comparability issues due to the use of different reference databases, and difficulties in processing the raw reads of this sequencer. In contrast, the MiSeq sequencer of Illumina used in our study has much lower error rates in sequencing and is the most widely available short-read sequencer. Furthermore, the LoopGenomics pipeline conducts the pre-processing into full-length reads, which is easy to use. Next, the reads are identified using the SILVA database as a reference for both short and full-length sequences, which currently is one of the best databases available for 16S rRNA sequences [26]. In addition, as previously stated, not only bacteria but also eukaryotic and archaeal microorganisms are discovered due to the primer mix included. This might be of importance for further studies that want to assess the impact of yeast-associated mastitis, such as those performed by previous studies, which found that even though yeast-associated mastitis is rare, it could be of importance for some clinical cases of intramammary infections [34,50]. Besides this, the species-level identification of milk-associated microorganisms is of great interest for animal husbandry and dairies in general.

One further example in this mentioned respect is the identification and differentiation of *Pseudomonas* spp., which are often associated with milk spoilage [51]. In our study, using the LoopSeq approach, we could identify *P. brenneri*, *P. canadensis*, *P. fluorescens*, *P. gessardii*, *P. helleri*, *P. lundensis*, *P. mucidolens*, *P. pseudoalcaligenes*, *P. putida* and *P. rhizosphaerae*, some of which are known to be proteolytic *Pseudomonas* spp. that occur frequently in retail milk [52]. In contrast, short amplicon data were not useful for the identification of *Pseudomonas* at the species-level, neither in V1–V2 nor in V3–V4 data, with only a questionable *Pseudomonas lurida*, because this species is not found in the full-length data. Most probably, this is a misclassification corresponding to *P. fluorescens* identified in the LoopSeq data, as *P. lurida* and *P. fluorescens* differ by only one nucleotide in the V3–V4 part of their 16S rRNA genes. 

## 5. Conclusions

Using the 16S/18S LoopSeq kit suitable for Illumina sequencing, we could not only identify bacteria at the species level but also the archaeal and eukaryotic microorganisms present in raw milk samples. The number of eukaryotic and archaeal reads varied from sample to sample, accounting for up to over 50% of all of the reads. Obviously, the bovine milk microbiome is highly diverse and different from sample to sample [7,12,53], which is reinforced by our study. The advantage of full-length SSU rRNA gene sequencing over short amplicon sequencing approaches is an improved species-level classification, as well as the simultaneous analysis of not only the bacteria present in the sample but also the identification of archaeal and eukaryotic species. Moreover, the LoopSeq kit as a commercial product allows for easy standardization across labs, and the downstream pipelines allow simple and convenient analysis with little bioinformatic knowledge required from the user. 

## Figures and Tables

**Table 1:** Characteristics of the bovine raw milk samples used in this study.

Sample Name	Total Bacterial Count (CFU/mL)	Individual Bacterial Count (IBC/mL)	Somatic Cell Count (SCC) per mL (Average Values Detected for Last 12 Months)
796	6.00 × 10^4^	2.23 × 10^5^	not available
797	1.23 × 10^5^	4.69 × 10^5^	not available
798	1.55 × 10^5^	5.97 × 10^5^	not available
879	5.30 × 10^4^	1.95 × 10^5^	247,000
880	2.70 × 10^4^	9.80 × 10^4^	149,000
978	2.90 × 10^4^	1.02 × 10^5^	146,000
979	2.30 × 10^4^	8.20 × 10^4^	90,000
980	2.00 × 10^4^	7.10 × 10^4^	185,000
981	3.20 × 10^4^	1.15 × 10^5^	91,000
983	9.00 × 10^3^	3.20 × 10^4^	96,000

**Table 2:** Top five genera/species detected in the raw milk samples for archaea and eukaryotes.

Top Hit	*Archaea*	*Eukaryotes*
Top 1	*Methanobrevibacter* sp.	*Pichia scutulata*
Top 2	*Methanobrevibacter millerae*	*Saprochaete clavata*
Top 3	*Methanosarcina soligelidi*	*Pichia cactophila*
Top 4	*Methanosarcina mazei*	*Kluyveromyces marxianus*
Top 5	*Methanosarcina horonobensis*	*Pichia pseudocactophila*

**Table 3:** Detection of species suspected to be mastitis-causing. The species are adapted from Cobirka et al. [20].

Species	Found in Raw Milk Samples	Detected on Genus Level in Raw Milk Samples	Found in Mock Communities
*Arcanobacterium/* *Truperella pyogenes*	yes	*Trueperella*	no
*Corynebacterium bovis*	no	*Corynebacterium*	no
*Enterobacter aerogenes*	no	no	no
*Enterococcus durans*	yes	*Enterococcus*	no
*Enterococcus faecalis*	yes		no
*Enterococcus faecium*	yes		no
*Escherichia coli*	yes	*Escherichia/Shigella*	*Escherichia coli*
*Klebsiella oxytoca*	yes	*Klebsiella*	no
*Klebsiella pneumoniae*	no		*Klebsiella pneumoniae*
*Mycoplasma bovis*	no	no	no
*Proteus* spp. ^(*)^	no	no	no
*Pseudomonas aeruginosa*	no	*Pseudomonas*	*Pseudomonas aeruginosa*
*Serratia marcescens* ^(*)^	yes	*Serratia*	no
*Staphylococcus aureus*	yes	*Staphylococcus*	*Staphylococcus aureus*
*Staphylococcus chromogenes*	yes		no
*Staphylococcus epidermidis*	yes		*Staphylococcus epidermidis*
*Staphylococcus haemolyticus*	yes		no
*Staphylococcus sciuri*	yes		no
*Staphylococcus simulans*	yes		no
*Streptococcus agalactiae*	yes	*Streptococcus*	no
*Streptococcus bovis*	no		no
*Streptococcus dysgalactiae*	yes		no
*Streptococcus equinus*	yes		no
*Streptococcus uberis*	yes		no
*Yersinia* spp. ^(*)^	no	no	no

* As, e.g., all *Serratia* spp. are listed according to Cobirka et al. [20], all of the species of this genus were considered to be possible mastitis-causing bacteria.

## Data Availability

The raw sequencing data (for the short amplicon sequencing) and the de-multiplexed fastq files of the contigs (for LoopSeq) are available at the Sequence Read Archive within the BioProject PRJNA719984.

## Supplementary Materials

The Supplementary Materials can be found at https://www.mdpi.com/article/10.3390/microorganisms9061251/s1. Table S1: PCR condition for the first step short amplicon 16S PCR. Table S2: PCR condition for the step short amplicon 16S PCR. Table S3: 16S rRNA gene short amplicon primer sequences. Table S4: PCR condition for LoopSeq Enrichment PCR. Table S5: List of the complete taxonomy of microorganisms detected within the milk samples processed using the full-length SSU rRNA gene sequencing approach (Excel table).
