# Supplementary material for: Full-Length SSU rRNA Gene Sequencing Allows Species-Level Detection of Bacteria, Archaea, and Yeasts Present in Milk
Source: Microorganisms. 2021 Jun 9;9(6):1251. doi: 10.3390/microorganisms9061251 (PMC8229006; doi:10.3390/microorganisms9061251)
Supplement: Supplementary file 1 [file microorganisms-09-01251-s001.zip › microorganisms-1244378-SI.pdf]

Supplementary Materials

Table S1. PCR condition for 1st step short amplicon 16S PCR.

PCR step		Temperature		Time	Step
Initial Denaturation		98 °C		2 min	1 cycle
Denaturation		98 °C		10 s	
Annealing	V1V2:	57 °C	milk:	30 s	20 cycles
	V3–V4:	55 °C	mock:	30 s	15 cycles
Extension		72 °C	milk:	90 s	
		72 °C	mock:	40 s	
Final Extension		72 °C		2 min	1 cycle
Storage		4 °C		hold	

Table S2. PCR condition for 2nd step short amplicon 16S PCR.

PCR step	Temperature	Time	Step
Initial Denaturation	98 °C	40 s	1 cycle
Denaturation	98 °C	20 s	
Annealing	55 °C	40 s	10 cycles
Extension	72 °C	40 s	
Final Extension	72 °C	2 min	1 cycle
Storage	4° C	hold	

Table S3. 16S rRNA gene short amplicon primer sequences.

Targeted region	Forward primer (5′–3′)	Reverse primer (5′–3′)	Reference
V1–V2	AGA GTT TGA TYM TGG CTC AG	GCT GCC TCC CGT AGG AGT	Salter, et al. [40]
V3–V4	CCT ACG GGN GGC WGC AG	GAC TAC HVG GGT ATC TAA TCC	Klindworth, et al. [52]

Table S4. PCR condition for LoopSeq Enrichment PCR.

PCR step	Temperature	Time	Step	Ramp speed
Initial Denaturation	95 °C	3 min	1 cycle	2 °C/s
Denaturation	98 °C	15 s		
Annealing	52 °C	20 s	30 cycles	2 °C/s
Extension	72 °C	2 min		
Storage	4 °C	hold		2 °C/s

Table S5. List of complete taxonomy of microorganisms detected within the milk samples processed using the full-length SSU rRNA gene sequencing approach (Excel table).
